# Book Reviews

**Published:** 1907-06

**Authors:** 


### BOOK REVIEWS.

---

PATHOGENIC MICRO-ORGANISMS—Including Bacteria and Protozoa. A practical manual for students, physicians and health officers, by William Hallock Park, M.D., Prof. of Bacteriology and Hygiene, University Hospital Medical College, and Director of the Research Laboratory of the department of Health, City of New York, assisted by Anna W. Williams, M.D., Assistnt Director of the Research Laboratory. Second edition, enlarged and thoroughly revised with 165 engravings and 4 full-page plates. Lea Bros. & Co., N. Y. and Phila.

The past few years have added greatly to our knowledge of the two classes of pathogenic micro-organisms, bacteria and protozoa, representing respectively the lowest forms of the vegetable and animal kingdoms. The importance of protozoa is now recognized, not only on account of the diseases known to be caused by them, but also on account of their possible conection with the exanthemata and syphilis.

The wide experience of Dr. Park and his prominence in this field of medicine assured success for his work from the first and the call for a second edition of his book was to be expected. It is sufficient to say that it is excellent in every detail and embodies our latest knowledge of the subject.

---

THE ESSENTIALS OF HISTOLOGY. Descriptive and practical for the use of students, by E. A. Schafer, LL.D., Sc.D., F.R.S., Prof. of Physiology in the University of Edinburgh, etc. Seventh edition. Lea Bros. & Co., Philadelphia and New York.

This well known book, now in its seventh edition is too well known to need an introduction to the medical profession as no doubt many of our readers used it as a text-book during their college days

This book, now in its seventh edition, is too well known to need an introduction to the medical profession as no doubt and well remember its clear, concise presentation of this subject. The present edition has been enlarged, especially that part dealing with the nervous system, both by additions to the text and by new illustrations. A new feature is the printing of many of the illustrations in color, which gives a better idea of the appearance of the stained specimens.

The book may be well taken as an ideal one for students, as it comprises the essential facts of the science, but omits less important details.

---

THE PRACTICE OF PEDIATRICS. In original contributions by American and English authors, edited by Walter L. Carr, A.M., M.D. Consulting physician to the French Hospital, New York, to the New York Eye and Ear Infirmary; visiting physician to the New York children's hospitals and schools, etc. Illustrated with 199 engravings and 32 full-page plates. Published by Lea Brothers & Co., Philadelphia and New York.

This excellent treatise upon pediatrics comprises the most up-to-date views by men of much prominence and experience and is one of the best books we possess in this line of medicine. Beginning with the normal infant and the injuries of the new born, it takes up the growth, development and hygiene and passes to infant feeding, all of which subjects are discussed in a logical scientific manner. Section II is composed of five chapters upon diseases of the alimentary tract. Section V is on diseases of nutrition by George M. Tuttle. Section VI is devoted to the infectious diseases. Other equally important sections are on the diseases of the respiratory tract, heart and blood vessels, genito-urinary systm, blood, lymphatic system, nervous diseases and those of the skin. With text and index there are 1014 pages of valuable literature, modern in character and well arranged.

The men who have contributed are: Abt, Bovaird, Crandall, Dade, Davis, Jennings, McCarthy, Nicoll, Poynton, Riviere, Ruhrah, Southworth Tuttle and Yale.

